# Metronomic delivery of orally available pemetrexed-incorporated colloidal dispersions for boosting tumor-specific immunity

**DOI:** 10.1080/10717544.2021.1995077

**Published:** 2021-11-03

**Authors:** Ruby Maharjan, Laxman Subedi, Rudra Pangeni, Saurav Kumar Jha, Seo Hee Kang, Kwan-Young Chang, Youngro Byun, Jeong Uk Choi, Jin Woo Park

**Affiliations:** a Research Institute of Pharmaceutical Sciences, College of Pharmacy, Seoul National University, Seoul, South Korea; b Department of Biomedicine, Health & Life Convergence Sciences, BK21 Four, Biomedical and Healthcare Research Institute, Mokpo National University, Muan-gun, South Korea; c Department of Pharmaceutics, School of Pharmacy, Virginia Commonwealth University, Richmond, VA, USA; d Global R&D Center, IcureBNP, Seoul, South Korea; e College of Pharmacy, Chonnam National University, Gwangju, South Korea; f College of Pharmacy, Natural Medicine Research Institute, Mokpo National University, Muan-gun, South Korea

**Keywords:** Pemetrexed, colloidal dispersion, oral metronomic chemotherapy, immunogenic cell death, antitumor immunity, immunotherapy

## Abstract

In this study, we developed oral pemetrexed (PMX) for metronomic dosing to enhance antitumor immunity. PMX was electrostatically complexed with positively charged lysine-linked deoxycholic acid (DL) as an intestinal permeation enhancer, forming PMX/DL, to enhance its intestinal permeability. PMX/DL was also incorporated into a colloidal dispersion (CD) comprised of the block copolymer of poly(ethylene oxide) and poly(propylene oxide), and caprylocaproyl macrogol-8 glycerides (PMX/DL-CD). CD-containing PMX/DL complex in a 1:1 molar ratio [PMX/DL(1:1)-CD] showed 4.66- and 7.19-fold greater permeability than free PMX through the Caco-2 cell monolayer and rat intestine, respectively. This resulted in a 282% improvement in oral bioavailability in rats. In addition, low-dose metronomic PMX led to more immunogenic cell death in CT26.CL25 cells compared to high PMX concentrations at the maximum tolerated dose. In CT26.CL25 tumor-bearing mice, oral metronomic PMX/DL-CD elicited greater antitumor immunity not only by enhancing the number of tumor-infiltrating lymphocytes but also by suppressing T cell functions. Oral PMX/DL-CD substantially increased programmed cell death protein ligand-1 (PD-L1) expression on tumor cells compared to the control and PMX-IV groups. This increased antitumor efficacy in combination with anti-programmed cell death protein-1 (aPD-1) antibody in terms of tumor rejection and immunological memory compared to the combination of PMX-IV and aPD-1. These results suggest that oral metronomic scheduling of PMX/DL-CD in combination with immunotherapy has synergistic antitumor effects.

## 1. Introduction

Pemetrexed (PMX) is a prototype anticancer chemotherapeutic that inhibits folate pathways and is a component of the standard treatment regimen for non-small cell lung cancer (NSCLC). A combination of PMX and cisplatin has been approved as first-line therapy for NSCLC. However, despite high therapeutic efficacy, acquired resistance and high recurrence rates lead to low overall survival rates in NSCLC patients (Darvin et al., 2018; Ng et al., 2019). A combination of conventional therapy and immune checkpoint inhibitors (ICIs), such as pembrolizumab and nivolumab, has recently been recommended as an alternative first-line therapy for NSCLS, and numerous clinical trials investigating combination therapies are in progress (Garon et al., 2019; Reck et al., 2019; Wu et al., 2020). Some of these clinical trials have demonstrated synergistic effects of combination therapies.

Conventional chemotherapy is administered at the maximum tolerated dose (MTD), leading to several adverse effects, including tumor resistance, neutropenia, myelosuppression, etc. To overcome these adverse effects, a drug-free period is scheduled, during which blood vessel regrowth and tumor recurrence can occur. As an alternative, low-dose metronomic chemotherapy (MCT) without drug-free periods was introduced, to maintain constant plasma drug levels and prevent blood vessel regrowth, and to achieve total remission with less toxicity (Simsek et al., 2019). MCT targets circulating endothelial and endothelial progenitor cells to prevent tumor angiogenesis (André et al., 2014). Furthermore, MCT has evolved into a multi-targeted therapy with immunomodulatory and tumor dormancy-inducing effects (Gnoni et al., 2015; Chen & Mellman, 2017). It boosts immunity via induction of immunogenic cell death (ICD), promotion of antigen presentation by dendritic cells, and downregulation of suppressive cells to increase the cytotoxic effects of effector immune cells (Ghiringhelli et al., 2007; Banissi et al., 2009; Hao et al., 2014). MCT is also associated with reduced immunotoxicity, where severe lymphocyte toxicity is seen with the MTD (Wu & Waxman, 2018). The effects of immune-supportive drugs administered as low-dose MCT are further enhanced due to lower immunotoxicity. For frequent metronomic dosing, oral administration is required. However, most chemotherapeutics, including PMX, are not orally available, which is one of the limitations of applying the MCT. In addition, there are further limitations, including low volume of distribution and rapid clearance (Rinaldi et al., 1999; Hanauske et al., 2001; Soni et al., 2017).

Previously, to overcome these problems, an electrostatic complex of PMX was prepared using positively charged lysine-linked deoxycholic acid (DA) (i.e. DL) as an intestinal permeation enhancer, forming PMX/DL, followed by incorporation into water-in-oil-in-water (w/o/w) multiple nanoemulsions, which provided significant increases in the intestinal membrane permeability as well as oral bioavailability of PMX with equivalent antitumor activities compared to intravenous (IV) injection of PMX (Mahmud et al., 2018; Pangeni et al., 2018a, 2018b). However, to further improve the stability of PMX in the nanoemulsions, a solid oral powder formulation was designed by incorporation of PMX/DL with dispersing agents, such as Kolliphor P188 and Labrasol, to produce a colloidal dispersion (CD) of PMX/DL (PMX/DL-CD) (Maharjan et al., 2019; Pangeni et al., 2019). In addition, *in vitro* permeability and oral absorption of PMX/DL-CD in rats were significantly increased by apical sodium-dependent bile acid transporter (ASBT)-facilitated endocytosis, caveola/lipid raft-dependent endocytosis, macropinocytosis, and paracellular transport (Park et al., 2017; Pangeni et al., 2019). Furthermore, metronomic treatment of A549 xenograft mice using oral PMX/DL-CD exhibited enhanced antiangiogenic activities, resulting in greater tumor growth suppression effects compared to MTD-treated mice (Maharjan et al., 2019).

The present study was mainly performed to determine the optimum complexation ratio of PMX and DL, to enhance oral bioavailability, and identify immune-modulating activities of metronomic dosing of oral PMX/DL-CD, followed by synergistic immunogenic antitumor effects of the metronomic dosing schedule of oral PMX/DL-CD in combination with ICIs. In this study, the optimum complexation molar ratio of PMX and DL was determined based on the permeability through a Caco-2 cell monolayer and rat intestinal perfusion study. After confirming the dose-dependent effects of PMX/DL-CD on oral absorption in rats, its antitumor activity in a Lewis lung carcinoma (LLC) mouse model was assessed. Furthermore, the immune-stimulating effects of metronomic treatment with oral PMX/DL-CD were compared to those of IV PMX at the MTD via ICD induction in CT26.CL25 cells. Finally, the synergistic anticancer effects of daily oral PMX/DL-CD and anti-programmed cell death protein-1 (aPD-1) antibody were evaluated in a CT26.CL25 colon cancer model in terms of complete response (CR) and immunological memory.

## 2. Experimental design

### 2.1. Materials

PMX disodium was purchased from Qilu Pharmaceutical Co., Ltd. (Jinan, China). Poly(ethylene glycol)-block-poly(propylene glycol)-block-poly(ethylene glycol) (Kolliphor P188) was provided by BASF pharmaceuticals (Ludwigshafen, Germany). Caprylocaproyl macrogol-8 glycerides (Labrasol) were obtained from Gattefossé SAS (Saint-Priest, France). 2,4-Diamino-*N*,10-methylpteroic acid (4-[*N*-(2,4-diamino-6-pterinidinyl-methyl)-N-methylamino]benzoic acid hemihydrochloride hydrate; DAMPA), 4′,6-diamidino-2-phenylindole (DAPI), DNAse, dispase, and histopaque-1077 were purchased from Sigma-Aldrich (St. Louis, MO, USA). Solvents for high-performance liquid chromatography (HPLC) and liquid chromatography-tandem mass spectrometry (LC-MS) analyses were obtained from the Merck group (Darmstadt, Germany) and Thermo Fisher Scientific (Waltham, MA, USA). The fluorescence-conjugated CD45, CD3, CD4, CD8, PD-L1, ki67, and IFN-γ antibodies were purchased from Biolegend (San Diego, CA, USA). Anti-CD16/CD32 antibody (Fc blocker) was purchased from BD Biosciences (San Jose, CA, USA). Calreticulin (CRT) antibodies and high mobility group box-1 (HMGB-1) were purchased from Abcam (Cambridge, UK). Texas Red-X conjugated wheat germ agglutinin solution, RIPA buffer, and collagenase were purchased from Thermo Fisher Scientific.

### 2.2. Animals

Sprague–Dawley (SD) rats (males, 6–7 weeks old, 200–250 g), C57BL/6 mice (females, 6–7 weeks old, 20–25 g), and BALB/c mice (females, 6–7 weeks old, 20–25 g) were purchased from G-Bio (Gwangju, South Korea). The animals were housed under standard housing conditions (temperature: 23 ± 2 °C; relative humidity: 55 ± 10%; and 12/12-h light/dark cycle). The animals had *ad libitum* access to a standard laboratory diet (Nestlé Purina PetCare Company, St. Louis, MO, USA) and ion-sterilized tap water.

Ethical approval for this study was obtained from the Institutional Animal Care and Use Committee (IACUC) of Mokpo National University (Jeonnam, South Korea; approval nos. MNU-IACUC-2020-013, MNU-IACUC-2020-018, and MNU-IACUC-2021-001). All animal experiments were performed in accordance with the National Institute of Health Guidelines for the Care and Use of Laboratory Animals and IACUC guidelines.

### 2.3. Preparation and characterization of PMX/DL-CD

**Figure 1.**
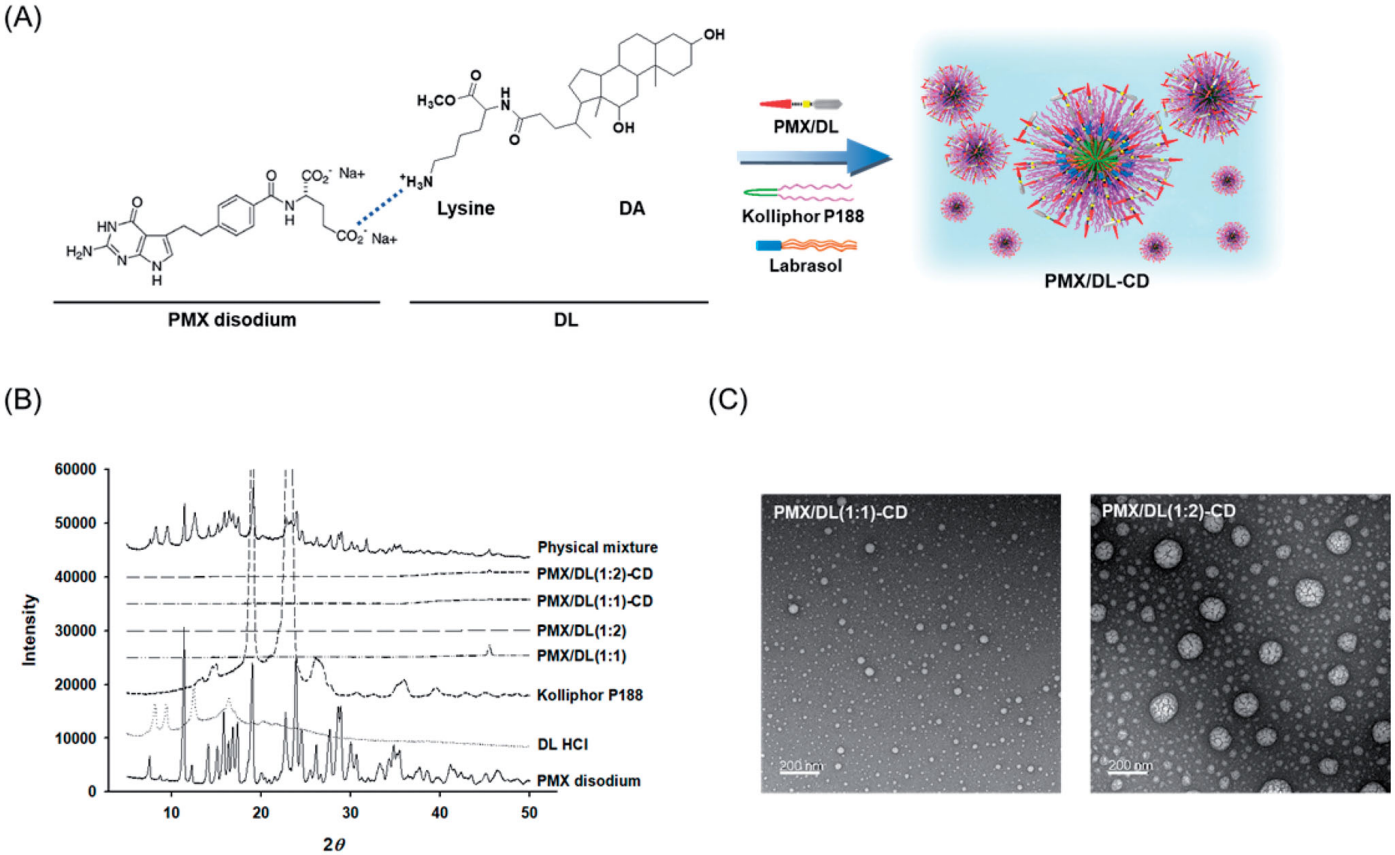
(A) Ion-pairing complex of PMX with DL and formation of the self-assembled nano-sized micellar structure of PMX/DL with Kolliphor P188 and Labrasol. (B) Powder X-ray diffraction (PXRD) of PMX disodium, DL·HCl, Kolliphor P188, PMX/DL(1:1), PMX/DL(1:2), PMX/DL(1:1)-CD, PMX/DL(1:2)-CD, and a physical mixture of PMX disodium, DL·HCl, and Kolliphor P188. (C) Transmission electron micrographs of PMX/DL-CD micelles. Scale bar: 200 nm.

Oral absorption enhancer (DL) was synthesized by the conjugation of DA with positively charged lysine, as described previously (Pangeni et al., 2018a; Choi et al., 2020). To prepare PMX/DL-CD, an aqueous solution of DL·HCl was then added, dropwise, to the aqueous solution of PMX disodium at molar ratios 1:1 and 1:2 with continuous stirring, forming PMX/DL(1:1) and PMX/DL(1:2), respectively. In addition, to prepare the CDs of PMX/DL(1:1) and PMX/DL(1:2), PMX disodium was dissolved in a 10% Kolliphor P188 solution containing 7.5% Labrasol, and then DL·HCl solution was added dropwise to form an ionic complex of PMX and DL with molar ratios of 1:1 and 1:2 [i.e. PMX/DL(1:1)-CD and PMX/DL(1:2)-CD, respectively] (Figure 1(A)). Furthermore, to improve the drug stability during storage, PMX/DL(1:1), PMX/DL(1:2), PMX/DL(1:1)-CD, and PMX/DL(1:2)-CD solutions were freeze-dried at −70 °C to make powdered forms.

To confirm the formation of ionic complexes, as well as CDs of PMX and DL with or without Kolliphor P188 and Labrasol, powder X-ray diffractions (PXRDs) of PMX, DL, Kolliphor P188, PMX/DL(1:1), PMX/DL(1:2), PMX/DL(1:1)-CD, PMX/DL(1:2)-CD, and physical mixture of PMX, DL, and Kolliphor P188 were performed. The PXRD diffractograms were observed using the Empyrean X-ray diffractometer (Malvern Panalytical, Malvern, UK) operated at 40 mA and 40 kV using copper (Cu-Kα1) radiation (*λ* = 1.5418 Å). Powdered samples were packed on 0.5-mm thick adhesive supports and scanned using the step-scan mode in a 2-theta range of 5–50° at a scanning rate of 0.02° per second.

Moreover, the re-dispersed forms of PMX/DLs and CDs comprising the complexes were further characterized based on particle sizes, polydispersity indices (PDIs), and zeta potentials at 25 °C using a dynamic laser light scattering analyzer (Malvern Zetasizer Nano ZS90; Malvern Panalytical, Malvern, UK). Morphological evaluations were performed using high-resolution transmission electron microscopy (TEM) (JEM-200; JEOL Ltd., Tokyo, Japan) after negative staining with a 2% aqueous solution of phosphotungstic acid.

### 2.4. *In vitro* Caco-2 cell monolayer permeability of PMX/DL-CD

To compare the *in vitro* apparent permeability (*P*_app_) of PMX, PMX/DL(1:1), PMX/DL(1:2), PMX/DL(1:1)-CD, and PMX/DL(1:2)-CD across a Caco-2 (ATCC^®^ HTB-37™; American Type Culture Collection, Manassas, VA, USA) cell monolayer, Caco-2 cells were seeded onto 24-well Transwell^®^ filter inserts in Dulbecco's modified Eagle's medium (DMEM) containing 10% (v/v) fetal bovine serum and 1% penicillin/streptomycin, at a density of 1 × 10^5^ cells/well, cultured for 14–16 days to form a confluent monolayer with the transepithelial electrical resistance (TEER) >350 Ω·cm^2^. After the media in the apical and basolateral compartments were replaced with Hank’s balanced salt solution (HBSS) and stabilized for 20 min at 37 °C, 100 µL of the drug solution diluted with HBSS (equivalent to 50 µg/mL PMX) and 600 µL of HBSS were loaded into the apical and basolateral compartments in each well, respectively. During incubation at 37 °C, 100 µL of sample solution was withdrawn from each basolateral compartment, and the volume was replaced using fresh HBSS at 0.5, 1, 2, 3, 4, and 5 h. After passing through a polyvinylidene fluoride (PVDF) membrane filter (pore size: 0.45 µm), the concentration of PMX in each sample solution was measured using the HPLC system at 254 nm, with a Luna C18 column (4.6 × 250 mm, 5 µm, 100 Å) at 25 °C. The mobile phase consisted of water (pH 3.5 adjusted with phosphoric acid) and acetonitrile (80:20, v/v), at a flow rate of 1 mL/min. The *P*_app_ of PMX from each formulation was estimated using the following formula: *P*_app_ = *dM*/*dt* × 1/(*S* × *C_i_*), where *dM*/*dt* indicates the linear-appearance permeation rate of PMX across a monolayer (μg/s), *C_i_* is the initial concentration of PMX in the apical compartment (μg/mL), and *S* is the permeation area of the monolayer (cm^2^).

### 2.5. *In situ* single-pass rat intestinal perfusion study

The *in situ* single-pass intestinal effective permeabilities (*P*_eff_) of PMX, PMX/DL(1:1), PMX/DL(1:2), PMX/DL(1:1)-CD, and PMX/DL(1:2)-CD were investigated in rats. The rats were fasted overnight for 12–16 h with free access to water before the experiment and then anesthetized by intramuscular injection of tiletamine·HCl–zolazepam mixture (1:1 [w/w]; 40 mg/kg). With the animal in the supine position, the abdominal cavity was opened using a midline longitudinal incision, and the intestine (duodenum, jejunum, and ileum) was exposed and subsequently intubated at the two ends with a polypropylene perfusion tube connected to a peristaltic pump (Harvard Apparatus, Holliston, MA, USA). After covering the entire excised area with a cotton pad soaked with warm normal saline, the cannulated intestinal segments were flushed with blank perfusion buffer at 37 °C for 30 min, at a constant flow rate of 0.5 mL/min, to remove residual intestinal contents. Then, drug solutions of PMX, PMX/DL(1:1), PMX/DL(1:2), PMX/DL(1:1)-CD, or PMX/DL(1:2)-CD (equivalent to 100 μg/mL PMX) were perfused through the intestinal lumen at 37 °C and a constant flow rate of 0.2 mL/min. After allowing 30 min to achieve a steady-state, outlet perfusate was collected every 15 min for 120 min. In addition, to assess the reliability of the perfusion study, 100 μg/mL fluorescein diluted in perfusion buffer was perfused separately under the same conditions and the collected samples were analyzed at excitation/emission wavelengths of 494/512 nm using a microplate reader (PerkinElmer multimode plate reader; PerkinElmer, Waltham, MA, USA) as a low-permeability marker. During the experiment, all animals were kept on heated pads under a heat lamp to maintain a normal body temperature. At the end of the experiment, the lengths and radii of the cannulated intestinal segments were measured, taking care to avoid stretching them. The collected samples were then filtered using 0.45-μm PVDF filters and stored at −20 °C before analyses. The outlet concentration of PMX in the perfusate was analyzed by HPLC as described above. In addition, the errors caused by rat intestinal water absorption and secretion during perfusion were corrected by the net water flux (ratio of the inlet to outlet perfusate flows, *Q*_out_/*Q*_in_) and the corrected drug concentration in perfusate for each formulation was calculated based on the following equation (i.e. gravimetric method): *C*_out,corr_ = *C_out_* × (*Q*_out_/*Q*_in_), where *C*_out,corr_ is the corrected outlet PMX concentration (µg/mL), *C*_out_ is the PMX outlet concentration (µg/mL), *Q*_in_ (mL/min) is the flow rate entering the intestine, and *Q*_out_ (mL/min) is the measured perfusate exit flow (net weight/15 min, assumed density of 1.0 g/mL) for the specified time interval. After correction, the *P*_eff_ was calculated using the equation: *P*_eff_ = *Q*_in_/*A* × *ln*(*C*_out,corr_/*C*_in_), where *A* is the intestinal area available for absorption (2π*rl*) based on its length (*l*) and radius (*r*).

### 2.6. *In vivo* pharmacokinetic study in rats

Oral absorption of PMX/DL(1:1)-CD was assessed to investigate the effects of ionic complex formation with DL, as well as CD formulations with Kolliphor P188 and Labrasol. Rats were orally administered 400 µL of PMX-Oral (20) (20 mg/kg PMX dissolved in water), PMX/DL(1:1)-CD (10) (equivalent to 10 mg/kg PMX), PMX/DL(1:1)-CD (20) (equivalent to 20 mg/kg PMX), and PMX/DL(1:1)-CD (40) (equivalent to 40 mg/kg PMX). Furthermore, to evaluate oral bioavailability, 150 µL of PMX-IV (10) (10 mg/kg PMX dissolved in water) was injected via the tail vein. Blood samples (150 µL) were drawn from the retroorbital plexus of rats at 0.5, 1, 1.5, 2, 4, 6, 8, and 10 h after oral administration or 0.25, 0.5, 1, 1.5, 2, 3, 4, and 8 h after IV injection under mild anesthesia. After mixing with 50 μL of 3.8% sodium citrate solution, the collected samples were immediately centrifuged at 2500 × *g* and 4 °C for 15 min. The separated plasma samples were kept at −80 °C before analysis and the drug concentration in each plasma sample was determined by LC-MS. Then, 10 µL of DAMPA (5 µg/mL) was added to each sample as an internal standard (IS) and subjected to solid-phase extraction using Plexa Bond Elut PAX cartridge (30 mg, 1 mL; Agilent Technologies, Santa Clara, CA, USA), conditioned with 500 µL of methanol, followed by 500 µL of deionized water. After loading the standards or samples into the cartridges, the unadsorbed particles were washed out with 500 µL of deionized water and methanol. Finally, the adsorbents were eluted with 250 µL of 5% formic acid in methanol twice and the eluent was then dried in a centrifugal evaporator (Genevac Ltd., Ipswich, UK). The dried eluate was then reconstituted with 100 µL of 5% formic acid in methanol and the concentration of PMX in each sample was determined using an Agilent 6120 Quadruple LC-MS system equipped with a Luna C18 column (100 × 2 mm, 3 µm). A solvent mixture consisting of acetonitrile and 0.34% formic acid solution (15:85, v/v) was used as the mobile phase, at a flow rate of 0.2 mL/min. PMX and IS were ionized using electrospray ionization (ESI) source in positive ion mode under a capillary voltage of 3.5 kV, drying gas flow rate of 3.1 L/min, and drying gas temperature of 300 °C. Quantification of the protonated molecular ions was done at ([M + H]^+^ = 428) and ([M + H]^+^ = 326.1) for PMX and DAMPA, respectively. In addition, the lowest limit of quantification (LOQ) and the limit of detection (LOD) of PMX were 5 ng/mL and 2.5 ng/mL, respectively.

### 2.7. *In vivo* antitumor efficacy of metronomic treatment with oral PMX/DL-CD in LLC cell-bearing mice

The *in vivo* dose-dependent antitumor effects of oral PMX/DL(1:1)-CD compared to PMX-IV were evaluated by inoculating LLC cells at a density of 1 × 10^6^ cells/100 μL PBS (pH 7.4) into the right dorsal flanks of 6-week-old female C57BL/6 mice. The oral doses for PMX/DL(1:1)-CD with antitumor activities without toxic effects were determined based on our previous study (Maharjan et al., 2019). After the tumor volume reached 60–80 mm^3^, the mice were randomly divided into six groups (*n* = 12 per group): control (untreated), PMX-IV (150) (once-every-3-weeks IV injection, 150 mg/kg PMX), PMX-Oral (20) (once-daily oral administration of PMX in water, 20 mg/kg PMX), PMX/DL(1:1)-CD (10) (once-daily oral administration of PMX/DL(1:1)-CD, equivalent to 10 mg/kg PMX), PMX/DL(1:1)-CD (20) (once-daily oral administration of PMX/DL(1:1)-CD, equivalent to 20 mg/kg PMX), and PMX/DL(1:1)-CD (40) (once-daily oral administration of PMX/DL(1:1)-CD, equivalent to 40 mg/kg PMX). The body weights and tumor volumes of all mice were measured every 3 days. Tumor volume was then calculated as *a*^2^ × *b* × 0.52, where *a* and *b* were the width and length of the tumor, respectively. The mice were sacrificed and the tumor masses were measured 21 days after drug administration.

### 2.8. Immunofluorescence analysis of ICD induction by treatment with PMX/DL

CT26.CL25 cells, cultured in coverslips at 1 × 10^4^ cell density, were treated with 300 µM DL, 300 µM PMX, or PMX/DL (equivalent to 300 µM PMX) for 48 h. The PMX dose was converted from the *C*_max_ of the pharmacokinetic profile in patients treated with 500 mg/m^2^ PMX-IV (Li et al., 2007). The cells were then washed with PBS, fixed in 10% formalin solution, and incubated with a cell membrane marker, Texas Red-X conjugated wheat germ agglutinin solution (5 µg/mL), for 10 min at room temperature. The cells were washed and permeabilized with 1% Triton-X solution for 40 min at room temperature. The cells were then incubated overnight with primary anti-CRT (1:200) or anti-HMGB-1 (1:200) antibodies at 4 °C after washing with PBS. On the following day, the cells were washed and incubated with secondary anti-rabbit Alexa Fluor 488 (1:400; Invitrogen; Thermo Fisher Scientific) for 1 h at room temperature and then stained with DAPI. Confocal laser scanning microscopy (TCS SP8; Leica Microsystems, Wetzlar, Germany) was used to obtain images.

### 2.9. Flow cytometric analysis of CRT exposure induced by PMX/DL treatment

For the detection of surface-released CRT, CT26.CL25 cells (1 × 10^5^ per well) cultured in 6-well plates were incubated with 300 µM DL, 300 µM PMX or PMX/DL(1:1) (equivalent to 300 µM PMX) for 48 h. The cells were then washed, detached, and incubated with anti-CRT antibodies (1:200) diluted in flow cytometry staining buffer (FACS buffer; eBioscience Inc., San Diego, CA, USA) at 4 °C for 1 h. They were then washed and incubated with APC crosslinked anti-rabbit IgG antibody (1:200; Thermo Fisher Scientific) for 30 min at 4 °C. Subsequently, the cells were washed and resuspended in FACS buffer and quantified using flow cytometry (FACS Aria II; BD Biosciences).

### 2.10. ELISA of HMGB-1 release after treatment with PMX/DL

CT26.CL25 cells (1 × 10^5^ per well) cultured in 24-well plates were treated with 300 µM DL, 300 µM PMX or PMX/DL (equivalent 300 µM PMX) for 48 h, and the cell supernatants were collected. HMGB-1 concentrations in the supernatants were determined using ELISA according to the manufacturer’s protocol (IBL International, Hamburg, Germany).

### 2.11. Immunoblotting of CRT and HMGB-1 after treatment with PMX/DL

CT26.CL25 cells (1 × 10^5^ per well) cultured in 6-well plates were treated with 300 µM DL, 300 µM PMX or PMX/DL (equivalent 300 µM PMX) for 48 h. The cells were then lysed in RIPA buffer with protease and phosphatase inhibitors (GenDEPOT, Barker, TX, USA). Each sample was loaded with 20 µg of protein and fractionated using SDS-PAGE (Biosesang, Gyeonggi-do, South Korea), followed by wet transfer to nitrocellulose membrane (Bio-Rad Laboratories, Inc., Hercules, CA, USA). Anti-CRT, anti-HMGB-1, GAPDH rabbit mAb (Cell Signaling Technology, Danvers, MA, USA), and anti-rabbit IgG peroxidase (Sigma–Aldrich) antibodies were used.

### 2.12. *In vivo* immunostimulatory effects of metronomic treatment with oral PMX/DL-CD

To investigate *in vivo* immunostimulatory effects of oral metronomic PMX, BALB/c mice were inoculated with 1 × 10^6^ of CT26.CL25 cells per mouse. The mice were randomly divided into three groups (10 mice per group) once the tumor volume had reached 50–70 mm^3^: control (untreated), PMX-IV (150 mg/kg PMX administered IV every 3 weeks), and PMX/DL-CD (oral PMX/DL(1:1)-CD administered daily, equivalent to 20 mg/kg PMX). The mice were sacrificed 3 weeks after treatment and the tumors were isolated. The tumor tissues were converted into single cells by passing them through a 40-µm filter after digestion with collagenase A (0.2% w/v), DNAse (30 U/mL), and dispase (10 U/mL) in MACS (gentleMACS Octo Dissociator with Heaters; Miltenyi Biotec, Bergisch Gladbach, Germany) at 37 °C for 45 min. Some cells were collected for tumor-infiltrating lymphocyte analyses and centrifuged using histopaque-1077 for 20 min at 450 g to isolate the lymphocytes. The isolated cells were then stained with fluorescence-conjugated antibodies and incubated. The cells were washed and suspended in FACS buffer and then collected into FACS tubes. The cells were then analyzed using flow cytometry (FACS Aria II) and the data were analyzed using FlowJo software (FlowJo LLC, Ashland, OR, USA). Cell populations were acquired by gating, as follows: tumor-infiltrating lymphocytes (DAPI^−^/CD45^+^), helper T cells (DAPI^−^/CD45^+^/CD3^+^/CD4^+^), PD-L1-expressing tumor cells (DAPI^−^/CD45^−^/PD-L1^+^), proliferating cytotoxic T cells (DAPI^−^/CD45^+^/CD3^+^/CD8^+^/ki67^+^), and IFN-γ-secreting T cells (DAPI^−^/CD45^+^/CD3^+^/CD8^+^/IFN-γ^+^).

Lymphocytes were isolated from the tumors using the procedure described previously; 2 × 10^5^ tumor lymphocytes per well, along with β-gal (5 µg/mL), were incubated on pre-coated 96-well plates in CTL test medium for 24 h at 37 °C with 9% CO_2_. The standard protocol for the Immunospot assay kit (Cellular Technology Ltd., Shaker Heights, OH, USA) was followed. After the procedure, the plates were scanned and the spots were counted in the ELISpot counter (Immunospot^®^ S6 ULTIMATE analyzer; Cellular Technology Ltd.).

Immunofluorescence was performed to investigate the expression of various immune cells in the tumor tissues of mice, inoculated and treated as previously described. The isolated tumor tissues were fixed with zinc fixation solution (BD Biosciences), embedded in paraffin, cut into 4-µm sections, and loaded into slides. The slides were deparaffinized using xylene, rehydrated in a series of alcohol solutions, and incubated in citrate buffer (GenDEPOT) at 100 °C for 60 min to retrieve the antigens. Slides were incubated with dye-conjugated CD45, CD8, F4/80, and PD-L1 antibodies overnight at 4 °C, and the nuclei were stained with DAPI. An automated, multimodal tissue analysis system (Vectra; PerkinElmer) was used to obtain images.

### 2.13. *In vivo* antitumor efficacy of oral PMX/DL(1:1)-CD and aPD-1 combination

To investigate the antitumor efficacy of oral PMX/DL(1:1)-CD and IV PMX given in combination with intraperitoneal aPD-1, 1 × 10^6^ of CT26.CL25 cells in 100 µL of PBS (pH 7.4) were subcutaneously inoculated into each BALB/c mouse. When the average tumor volume reached 50–70 mm^3^, mice were randomly divided into seven groups (10 mice per group): control (untreated), PMX-IV (150) (IV administration of 150 mg/kg PMX once every 3 weeks), aPD-1 (10) (intraperitoneal administration of 10 mg/kg aPD-1 once every 3 days), PMX-Oral (20) (once-daily oral administration equivalent to 20 mg/kg PMX), PMX/DL-CD (20) [once-daily oral administration PMX/DL(1:1)-CD equivalent to 20 mg/kg PMX], PMX-IV (150) + aPD-1 (10) (combination of IV administration of 150 mg/kg PMX once every 3 weeks, and intraperitoneal administration of 10 mg/kg aPD-1 once every 3 days), and PMX/DL-CD (20) + aPD-1 (10) (a combination of once-daily oral administration of PMX/DL(1:1)-CD equivalent to 20 mg/kg PMX and intraperitoneal administration of 10 mg/kg aPD-1 once every 3 days). During treatments, body weights and tumor volumes of mice in all seven groups were monitored once every 3 days, for 27 days. Tumor volumes in each mouse were calculated based on the following formula: 0.52 × *a*^2^ × *b*, where *a* and *b* were the tumor widths and lengths, respectively. To address the immunological memory response, mice showing complete remission were rechallenged subcutaneously with 1 × 10^6^ of CT26.CL25 cells in 100 µL of PBS (pH 7.4) on the opposite flank 30 days after the initial drug treatment. Then, tumor volumes and body weights of the mice were observed once every 3 days, as described earlier, without drug treatment.

### 2.14. Pharmacokinetics and statistical analyses

Pharmacokinetic parameters were estimated using a non-compartmental model in WinNonlin^®^ software (ver. 5.3; Pharsight Corp., Mountain View, CA, USA). All data were expressed as means ± standard deviations (SDs), or standard errors of the mean (SEMs). Statistical analysis was performed by one-way analysis of variance (ANOVA) followed by Tukey’s multiple comparison test for unpaired data with more than three means. In all analyses, *p* < .05 was taken to indicate statistical significance.

## 3. Results and discussion

### 3.1. Characterization of PMX/DL-CD

To confirm the ionic complex formation between PMX and DL, as well as to define the physical state of PMX/DL in the oral formulations, PXRD analyses were performed. Pure PMX disodium showed characteristic crystalline peaks at 7.5, 11.4, 12.1, 14.1, 15.1, 15.8, 16.3, 16.8, 17.3, 19.0, 22.7, 23.9, 24.5, 26.1, 27.7, 28.6, 28.9, 30.0, 30.6, 33.2, and 34.8 over the 2-theta range in the PXRD spectra (Figure 1(B)). In addition, DL·HCl exhibited crystalline peaks at 8.1, 9.4, 12.5, and 16.4 in the PXRD spectra (Figure 1(B)). The characteristic crystalline signals for PMX and DL were also detectable in a physical mixture of PMX disodium, DL·HCl, and Kolliphor P188. However, after ion-pairing complexation with DL [i.e. PMX/DL(1:1) and PMX/DL(1:2)], the crystalline drug peaks disappeared, indicating that PMX becomes amorphous in the complexes (Figure 1(B)). Moreover, PMX in the CD formulations consisting of PMX/DL(1:1) or PMX/DL(1:2) with Kolliphor P188 and Labrasol showed no drug crystallinity (Figure 1(B)). These results suggest that PMX in PMX/DL and PMX/DL-CD may be molecularly dispersed with DL, Kolliphor P188, and Labrasol, existing in an amorphous state, which may improve partitioning of PMX into the epithelial membrane.

The construction of micelles was investigated after incorporation of PMX/DL with Kolliphor P188 and Labrasol by analysis of the particle sizes, PDIs, and zeta potentials of aqueous dispersions of PMX/DL(1:1)-CD and PMX/DL(1:2)-CD. The particle sizes of PMX/DL(1:1) and PMX/DL(1:2) were estimated as 1116 ± 394 and 176 ± 4.95 nm, respectively, which confirmed the formation of PMX/DL(1:2)-assembled micelles, driven by the higher amphiphilic properties of PMX/DL(1:2) molecules than those of PMX/DL(1:1) due to the presence of two hydrophobic DA moieties in the complex (Table 1). The particle sizes of PMX/DL(1:1)-CD and PMX/DL(1:2)-CD were 175 ± 2.01 and 193 ± 2.72 nm, with PDIs of 0.157 ± 0.019 and 0.187 ± 0.005, respectively (Table 1). The significantly reduced particle size with narrow PDI for PMX/DL(1:1)-CD compared to PMX/DL(1:1) may have been due to decreased critical micellar concentration and increased micelle formation of PMX/DL(1:1) by Kolliphor P188 and Labrasol, which are an amphiphilic block copolymer and surfactant, respectively, and can form self-assembled micelles at concentrations above their critical micellar concentrations, assisting in the formation of well-arranged and compact nano-sized micelles comprising PMX/DL(1:1). In addition, the zeta potentials of PMX/DL(1:1)-CD and PMX/DL(1:2)-CD were 12.5 ± 0.850 and 19.3 ± 0.557 mV, which were 36.8 and 56.8 times higher than those of PMX/DL(1:2), respectively, ensuring the formation of stable dispersions due to electrostatic repulsion. Furthermore, TEM demonstrated uniform dispersion of PMX/DL complex in CD. The images for PMX/DL-CDs showed quasi-spherical micelles, homogeneous in size, <200 nm in diameter, without aggregation (Figure 1(C)).

**Table 1.** Properties of different PMX forms.

Test material	Particle size (nm)	Polydispersity index (PDI)	Zeta potential (mV)	Apparent permeability (*P*_app_, ×10^−6^ cm/s)	Effective permeability (*P*_eff_, ×10^−4^ cm/s)
PMX				1.20 ± 0.349	0.830 ± 0.044
PMX/DL(1:1)	1116 ± 394	0.878 ± 0.109	16.0 ± 0.954	3.24 ± 0.314***	2.71 ± 0.908
PMX/DL(1:2)	176 ± 4.95	0.397 ± 0.014	0.340 ± 0.290	4.76 ± 0.595***^,###^	3.27 ± 0.964
PMX/DL(1:1)-CD	175 ± 2.01	0.157 ± 0.019	12.5 ± 0.850	5.59 ± 0.409***^,###,$^	5.97 ± 1.26**
PMX/DL(1:2)-CD	193 ± 2.72	0.187 ± 0.005	19.3 ± 0.557	6.09 ± 0.724***^,###,$$$^	6.92 ± 3.36**^,#^

Apparent permeability (*P*_app_) and effective permeability (*P*_eff_) values of PMX with or without DL, Kolliphor P188, and Labrasol alone or in combination through a Caco-2 cell monolayer (*n* = 7) and the rat intestine (*n* = 4). Values represent means ± SDs.

**p < .01, ***p < .001 compared to PMX.

^#^*p* < .001, ^###^*p* < .001 compared to PMX/DL(1:1).

^$^*p* < .05; ^$$$^*p* < .001 compared to PMX/DL(1:2).

### 3.2. *In vitro* permeability of PMX/DL-CD through a Caco-2 cell monolayer

The absorption potentials of PMX/DLs and PMX/DL-CDs across a Caco-2 cell monolayer were assessed after preparation of ion-pairing complexes between PMX and DL at molar ratios of 1:1 and 1:2. The *P*_app_ of PMX/DL increased with an increasing complexation molar ratio of DL to PMX. PMX/DL(1:2) showed a 1.47-fold higher permeability than PMX/DL(1:1), resulting in a 297% increase in the *P*_app_ of free PMX (Table 1). This increase in *P_app_* was attributed to the DL in the complex, which renders the PMX molecule amphiphilic and improves its partition coefficient (Table S1) (Moghimipour et al., 2015). After oral formulation of PMX/DL with Kolliphor P188 and Labrasol, the permeability of PMX from PMX/DL(1:1)-CD or PMX/DL(1:2)-CD increased 1.73- and 1.28-fold compared to PMX/DL(1:1) and PMX/DL(1:2), and resulted in increases of 366% and 408% in the *P*_app_ of free PMX, respectively. The *P*_app_ of PMX formulated only with Kolliphor P188 and Labrasol (i.e. PMX-CD) was 2.07 ± 0.482 (×10^−6 ^cm/s), which was 1.73-fold higher than that of free PMX but 1.57- and 2.70-fold lower than those of PMX/DL(1:1) and PMX/DL(1:1)-CD, respectively. These results may have been due to the synergistic effects of DL and surfactants in PMX/DL-CD comprising the nano-micelles on their *trans*- or *para*-cellular permeation (Miyake et al., 2006; Moghimipour et al., 2015; Tian et al., 2017). The ionically conjugated DL in PMX/DL has been shown to facilitate penetration of a cargo molecule (i.e. PMX) through the cell membrane by ASBT (Pangeni et al., 2019; Deng & Bae, 2020). In addition, DL, as well as Kolliphor P188 and Labrasol, can increase membrane flexibility to increase drug partitioning across the membrane (Gupta et al., 2013; DiMarco et al., 2017; Pavlović et al., 2018; Jha et al., 2020) DA in DL, as well as surfactants in CD, can reversibly open the tight junctions by autophosphorylation of epidermal growth factor receptor (EGFR) or dephosphorylation and rearrangement of zonula occludens-1 (ZO-1), which may facilitate the permeation of PMX or PMX/DL released from PMX/DL-CD through the cell membrane via the paracellular pathway (Raimondi et al., 2008; Stojančević et al., 2013; Zhou et al., 2013; Pavlović et al., 2018). Moreover, PMX/DL-CD is known to be transported by caveola/lipid raft-mediated endocytosis and micropinocytosis (Kou et al., 2018; Pavlović et al., 2018; Pangeni et al., 2019). Overall, the combined activities of DL complex and CD formation with Kolliphor P188 and Labrasol may markedly improve the *P*_app_ of PMX/DL-CDs. However, the *P*_app_ of PMX/DL(1:1)-CD was not significantly enhanced by increasing the DL complexation ratio.

### 3.3. *In situ* single-pass intestinal perfusion of PMX/DL-CD in rats

The intestinal permeability of PMX was also investigated in rat intestinal segments using the *in situ* single-pass perfusion technique. After confirming the reliability of this study by measuring the *P*_eff_ of fluorescein (0.001 ± 0.002, ×10^−4 ^cm/s), the *P*_eff_ of PMX was 0.830 ± 0.044 (×10^−4 ^cm/s), which increased 3.27- and 3.94-fold after ion-pairing with DL at molar ratios of 1:1 and 1:2, respectively. In addition, oral formulation of PMX/DL(1:1)-CD exhibited significantly higher intestinal permeability (5.97 ± 1.26, ×10^−4 ^cm/s) compared to PMX/DL(1:1) and free PMX. The *P*_eff_ of PMX/DL(1:2)-CD was also 2.12- and 8.34-fold higher than those of PMX/DL(1:2) and free PMX, respectively (Table 1, Table S2). However, there were no significant differences in permeability between PMX/DL(1:1)-CD and PMX/DL(1:2)-CD. These results were consistent with the *in vitro* Caco-2 cell permeability data, which may have been induced by ASBT saturation (Kanda et al., 1998). Furthermore, the permeabilities of all formulations increased significantly in the perfusion study, possibly due to the greater available absorption area of the villi and microvilli (Dezani et al., 2016).

Based on the overall *in vitro* as well as *in situ* permeability data, PMX/DL(1:1)-CD was selected as the optimum oral PMX formulation. Further studies, including analyses of *in vivo* oral absorption and *in vivo* synergistic antitumor effects of metronomic dosing of oral PMX in combination with aPD-1, were carried out using PMX/DL(1:1)-CD.

### 3.4. *In vivo* pharmacokinetic study of PMX/DL-CD in rats

The plasma concentration-time curves and the pharmacokinetic parameters of PMX after IV and oral administration of PMX or PMX/DL(1:1)-CD at various doses in rats are presented in Figure S1 and Table 2. The maximum plasma concentration (C_max_) and area under the plasma concentration–time curve (AUC) achieved after oral administration of PMX/DL(1:1)-CD (10) (equivalent to 10 mg/kg PMX) were 1.81 ± 0.225 μg/mL and 7.02 ± 1.04 μg·h/mL, respectively, resulting in 358% higher bioavailability compared to oral PMX (20 mg/kg). Moreover, with an increasing dose of PMX/DL(1:1)-CD from 10 to 20 mg/kg, C_max_ (3.85 ± 0.371 μg/mL) the AUC (11.1 ± 1.71 μg·h/mL) increased significantly, 6.65- and 2.82-fold greater than oral PMX (20 mg/kg), respectively. Therefore, a single oral dose of PMX/DL(1:1)-CD (20) showed a significant increase in the oral bioavailability of PMX (22.0 ± 3.40%), which was 282% greater than that of PMX-Oral (20). In addition, the C_max_ and AUC values for PMX/DL(1:1)-CD (20) were 2.13- and 1.58-fold greater than those of PMX/DL(1:1)-CD (10) (equivalent to 10 mg/kg PMX), respectively. According to our previous studies regarding the intestinal transport mechanism of PMX/DL(1:1)-CD, ASBT-facilitated uptake was confirmed as a predominant pathway for oral absorption of PMX/DL(1:1)-CD (Pangeni et al., 2019). However, further increasing the dose of PMX/DL(1:1)-CD to 40 mg/kg (based on PMX) was associated with only a 1.62-fold increase in the oral bioavailability compared to free PMX (20). A 2-fold increase in the oral dose of PMX/DL(1:1)-CD (from 20 to 40 mg/kg based on PMX) yielded a C_max_ value of 5.07 ± 0.624 μg/mL, which was 1.32-fold higher than that of PMX/DL(1:1)-CD (20) (equivalent to 20 mg/kg PMX). Furthermore, the AUC for PMX/DL(1:1)-CD (40) also increased slightly from 11.1 ± 1.71 to 12.7 ± 2.70 μg·h/mL, resulting in 74.6% lower oral bioavailability than PMX/DL(1:1)-CD (20). This suggests that the oral absorption of PMX/DL(1:1)-CD increased in a dose-dependent manner. This non-linear relationship between dose and oral absorption of PMX/DL(1:1)-CD may reflect limitations in the intestinal membrane permeation due to ASBT saturation (Kanda et al., 1998; Pavlović et al., 2018; Deng & Bae, 2020).

**Table 2.** Pharmacokinetic parameters of PMX in rats after intravenous or oral administration of PMX or PMX/DL(1:1)-CD.

Test material	PMX-IV (10)	PMX-Oral (20)	PMX/DL(1:1)-CD (10)	PMX/DL(1:1)-CD (20)	PMX/DL(1:1)-CD (40)
Administration route	IV	Oral	Oral	Oral	Oral
PMX dose (mg/kg)	10	20	10	20	40
*T*_max_ (h)	–	0.50 ± 0.00	0.50 ± 0.00	0.50 ± 0.00	0.50 ± 0.00
*T*_1/2_ (h)	0.954 ± 0.043	13.1 ± 1.82	15.3 ± 1.18	13.9 ± 1.25	8.11 ± 1.91
*C*_max_ (μg/mL)	32.3 ± 1.22	0.579 ± 0.073	1.81 ± 0.225	3.85 ± 0.371	5.07 ± 0.624
AUC_last_ (μg·h/mL)	25.1 ± 3.56	3.93 ± 0.380	7.02 ± 1.04	11.1 ± 1.71	12.7 ± 2.70
AUC_inf_ (μg·h/mL)	25.3 ± 3.56	8.96 ± 0.089	19.7 ± 1.21	26.7 ± 4.65	20.7 ± 6.17
Bioavailability (%)	100	7.80 ± 0.755	27.9 ± 4.12	22.0 ± 3.40	12.6 ± 2.68

T_max_: time to reach the maximum plasma concentration of PMX; T_1/2_: plasma half-life of PMX; C_max_: maximum plasma PMX concentration; AUC_last_: area under the plasma concentration-time curve between zero and the last measurable plasma concentration; AUC_inf_: area under the plasma concentration-time curve between zero and infinity. Bioavailability (%) = (AUC_last, oral_/Dose_PMX, oral_)/(AUC_last, IV_/Dose_PMX, IV_) × 100. All values are means ± SDs (*n* = 4).

However, as the ion-pairing complex is less stable than the prodrug form, the possibility of premature complex dissociation in biological fluids before absorption is a major concern when utilizing the ion-pairing approach. On the other hand, the overall *in vitro* permeability and *in vivo* oral absorption data suggest that ion-pairing complexation with DL followed by formulation with surfactants, forming CDs, can deliver specific target molecules through the intestinal membrane more effectively via ASBT-mediated endocytosis and/or caveola/lipid raft-mediated endocytosis or micropinocytosis (Park et al., 2017; Pangeni et al., 2019), implying the formation of stable ion-pairing complexes were under physiological conditions after incorporation into micelles comprised of Kolliphor P188 and Labrasol. However, further studies to determine the stability and association constant of PMX/DL and its CD formulation in the gastric and intestinal fluids are required.

### 3.5. *In vivo* tumor growth inhibitory effect of metronomic oral PMX/DL-CD in LLC cell-bearing mice

**Figure 2.**
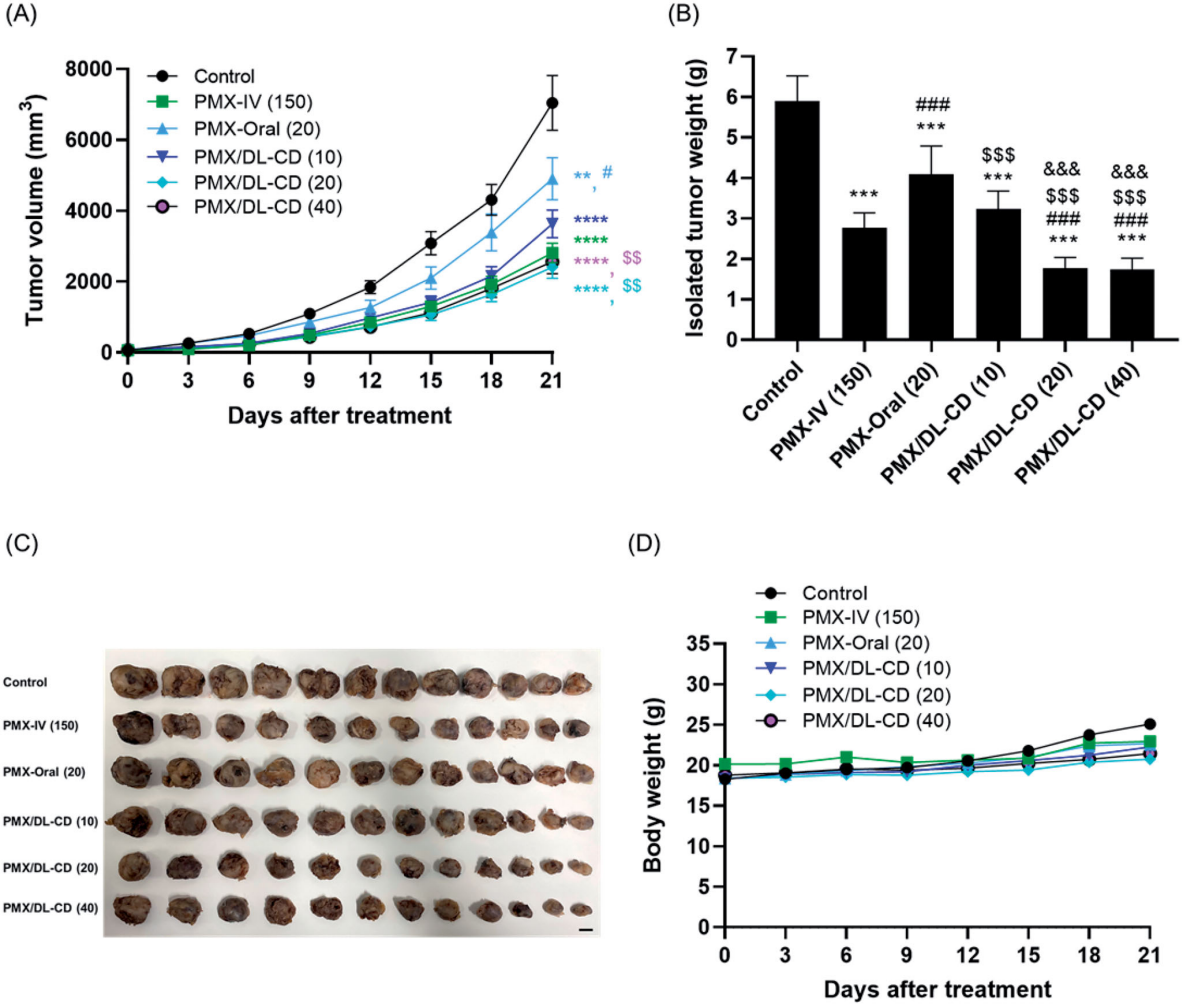
Efficacy of *in vivo* tumor-growth inhibitory effects of PMX in LLC tumor-bearing mice after intravenous injection of 150 mg/kg PMX: PMX-IV (150) once every three weeks, and once-a-day oral administration of aqueous solution of 20 mg/kg PMX: PMX-Oral (20) or PMX/DL(1:1)-CD as 10, 20, and 40 mg/kg PMX: PMX/DL-CD (10), PMX/DL-CD (20), and PMX/DL-CD (40), respectively, for 21 days. (A) Tumor growth inhibition in each group. (B) Excised tumor weights in LLC tumor-bearing mice. (C) Excised tumor tissues from each group on day 21. Scale bar: 10 mm. (D) Body-weight changes during treatment. All values are means ± SEMs (*n* = 12 for each group). **p < .01, ***p < .001, and ^****^p < .0001 compared to untreated controls; ^#^*p* < .05, and ^###^*p* < .001 compared to the PMX-IV (150) group; ^$$^*p* < .01, and ^$$$^*p* < .001 compared to the PMX-Oral (20) group; ^&&&^*p* < .001 compared to the PMX/DL-CD (10) group.

The dose-dependent tumor-growth inhibitory effects of oral PMX/DL-CD were investigated in the LLC cells-bearing mice model to assess the optimum oral dose for PMX/DL-CD. After once-a-day oral administration of PMX/DL-CD (10), the tumor growth was delayed by 50.3% compared to the control group (Figure 2(A)). In addition, PMX/DL-CD (10) exhibited a 1.34-fold higher tumor growth retardation rate compared to PMX-Oral (20), which indicates that enhanced permeability, followed by improved oral bioavailability may surpass the antitumor effects of oral PMX. As a daily oral dose of PMX/DL-CD was increased to 20 mg/kg based on PMX, the tumor volume was suppressed by 67.4%, compared to the control group. Moreover, PMX/DL-CD (20) delayed tumor growth by 50.9% compared to PMX-Oral (20), equivalent to the PMX-IV (150) group, representing a 60.9% antitumor efficacy compared to the control group. Thus, after 21-day treatment, the PMX/DL-CD (20) and PMX-IV (150) groups resulted in a 334% and 213% greater reduction in the isolated tumor weights, respectively, compared to the control group (Figure 2(B,C)). The greater tumor-suppressing ability of PMX/DL-CD (20), despite lower plasma levels compared to IV administration, might be due to the enhanced oral absorption of PMX from PMX/DL-CD (20), achieving the plasma and tumor-tissue levels necessary to elicit an anticancer response. Oral PMX/DL-CD (40) also resulted in a greater tumor volume suppression, by 69.6 and 54.3%, compared to the control and PMX-Oral (20) groups, respectively. However, there was no further increase in its anticancer effects, compared to the PMX/DL-CD (20) or PMX-IV (150)-treated mice. Furthermore, the isolated tumor weights were similar in mice treated with PMX/DL-CD (20). These findings were in agreement with the dose-dependent plasma levels of PMX. The C_max_ and AUC values for PMX/DL-CD (40) did not increase significantly and were two times greater than PMX/DL-CD (20). The body weights of mice treated with PMX or PMX/DL-CDs did not change significantly compared to the control group. This indicated that there were no additional toxic effects induced by the drug, or by oral formulations including DL, after repeated administration for 3 weeks (Figure 2(D)).

Collectively, these findings indicate that MCT of PMX/DL-CD (20) can deliver PMX effectively via the oral route, and can maintain sufficient drug levels in the tumor microenvironments to have significant antitumor efficacy compared to PMX-IV. Further *in vivo* synergistic anticancer efficacy studies of combined treatment with aPD-1 and oral PMX/DL-CD (20) were performed.

### 3.6. *In vitro* evaluation of ICD induction by treatment with PMX/DL

**Figure 3.**
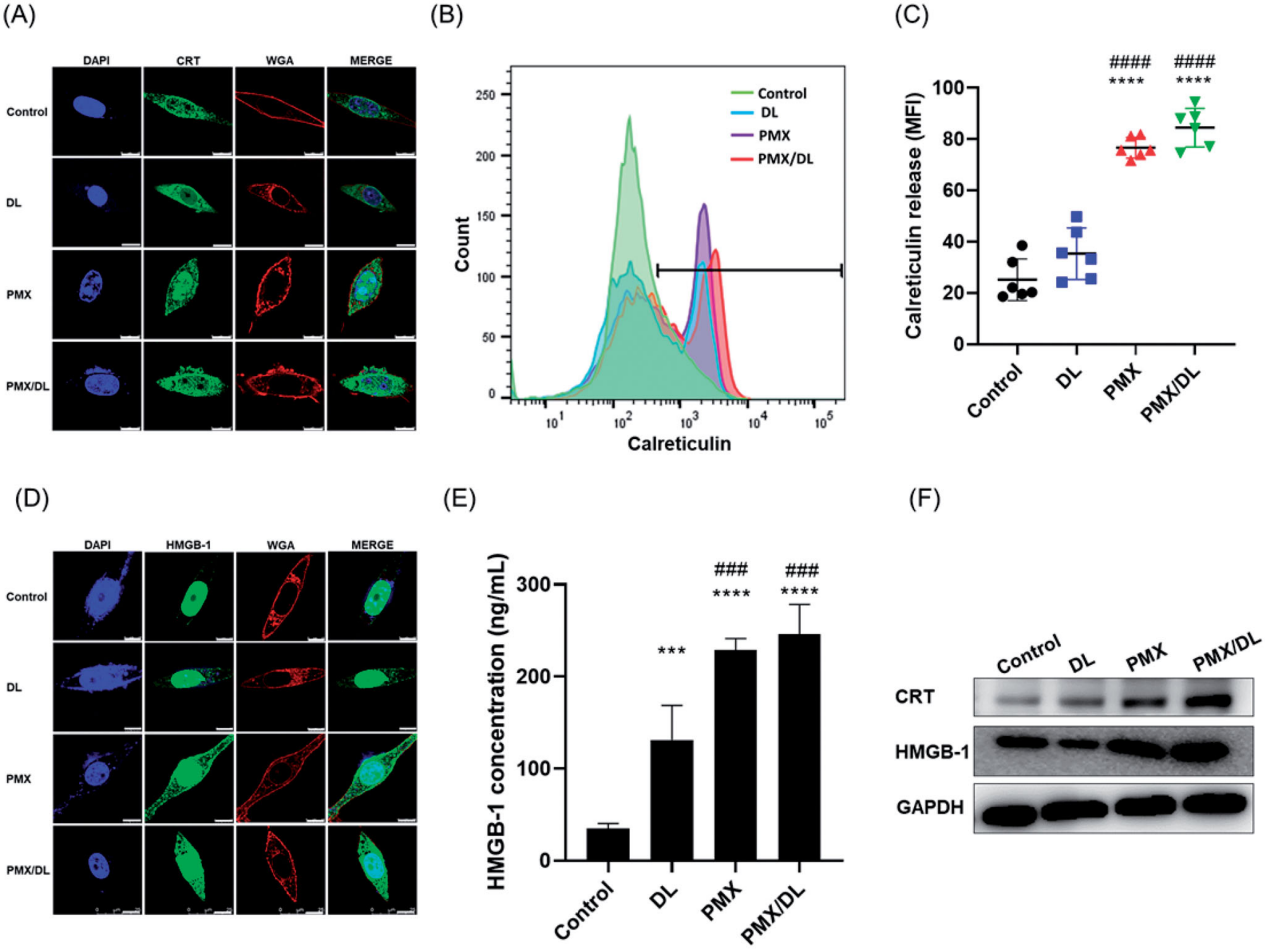
PMX-induced immunogenic cell death. CT26.CL25 cells were subjected to no treatment (control), DL, PMX, or PMX/DL. (A) Confocal micrograph of CT26.CL25 cells following treatment and DAPI staining of the nucleus (blue), and calreticulin (CRT, FITC), and wheat germ agglutinin (WGA; Texas Red) staining of the cell membrane. Scale bar: 10 μm. (B) *In vitro* analysis of cellular CRT release using flow cytometry. (C) Quantification of CRT release. (D) Confocal microscopy image of HMGB-1 release from cells. (E) Quantification of HMGB-1 release by ELISA. (F) Western blotting analysis of CRT and HMGB-1 release. All values are means ± SEMs (*n* = 12 in each group). ***p < .001, ****p < .0001 compared to controls; ^###^*p* < .001, ^####^*p* < .0001 compared to the DL treatment group.

ICD elicits an immune response characterized by translocation of CRT to the cell surface and subsequent HMGB-1-release from the nucleus. The release of CRT and HMGB-1 following PMX treatment was revealed in CT26.CL25 cells by confocal microscopy, flow cytometry, ELISA, and immunoblotting. Confocal microscopy showed that CRT-fluorescence was more intense in PMX-treated cells compared to controls and DL-treated cells, indicating greater CRT release with PMX treatment (Figure 3(A)). Increased CRT release in PMX-treated cells was also observed using flow cytometry. The surface CRT-release was 3.73, 2.36, and 1.11 times higher in PMX/DL-treated cells compared to untreated, DL-treated, and PMX-treated cells, respectively (Figure 3(B,C)). Similarly, HMGB-1-release was higher in PMX-treated cells compared to controls and DL-treated cells according to confocal microscopy (Figure 3(D)). This was verified by HMGB-1 ELISA, which demonstrated 7.04, 1.87, and 1.07 times higher HMGB-1 release in PMX/DL-treated cells compared to untreated, DL-treated, and PMX-treated cells, respectively (Figure 3(E)). Furthermore, western blot analysis of CT26.CL25 cells demonstrated higher CRT- and HMGB-1-protein content in PMX-treated compared to untreated and DL-treated cells (Figure 3(F)).

Taken together, the results suggested that PMX successfully induces ICD, which is key to the immune response. PMX/DL conjugate was a more potent ICD inducer than PMX alone, possibly due to increased cell permeability, as described previously.

### 3.7. Metronomic oral PMX/DL-CD treatment enhances both T cell population and function

**Figure 4.**
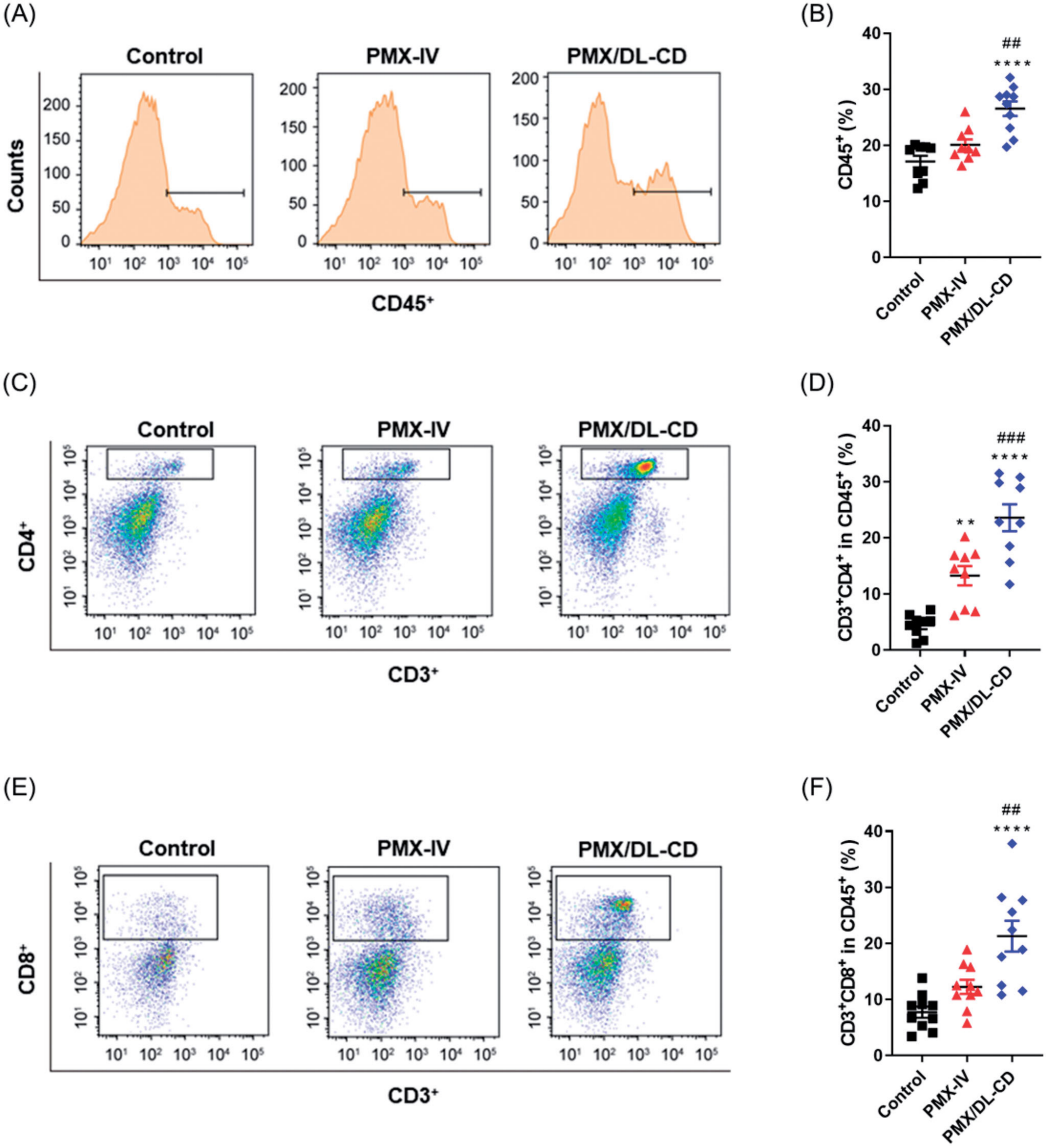
(A) Evaluation of total lymphocyte-tumor infiltration using flow cytometry and (B) quantification thereof in the control, MTD, and MCT groups. (C) Characterization of CD45^+^CD3^+^CD4^+^ T cells using flow cytometry and (D) quantification thereof. (E) Gating of CD45^+^CD3^+^CD8^+^ T cells using flow cytometry and (F) quantification thereof. All values are means ± SEMs (*n* = 5). **p < .01, and ^****^p < .0001 compared to untreated controls; ^##^*p* < .01, and ^###^*p* < .001 compared to the PMX-IV group.

As PMX provoked ICD in CT26.CL25 cells, we hypothesized that *in vivo* PMX treatment may promote antitumor immunity via tumor-infiltrating lymphocyte enhancement. To test this, we distinguished three groups: a control group, a group in which the MTD of PMX was injected into the tail vein (PMX-IV), and a metronomic oral PMX-treated (PMX/DL-CD) group. Tumor immune-modulatory effects were compared among the groups. The PMX-IV group had slightly increased CD45^+^-cell infiltration, while the PMX/DL-CD group had a significantly larger cell population compared to the control group (Figure 4(A,B)). Although the PMX-IV group had a substantially increased CD4^+^ T-cell population compared to the control group, the increase was even greater in the PMX/DL-CD group (Figure 4(C,D)). However, only the PMX/DL-CD group demonstrated an increase in CD8^+^ T-cell population compared to the control group (Figure 4(E,F)). These data suggest that although PMX treatment using MTD resulted in slightly elevated tumor-infiltrating lymphocytes, such as CD4^+^ T-cells, the overall antitumor immunity remained inadequate; metronomic oral PMX treatment could elicit much stronger antitumor adaptive immunity.

**Figure 5.**
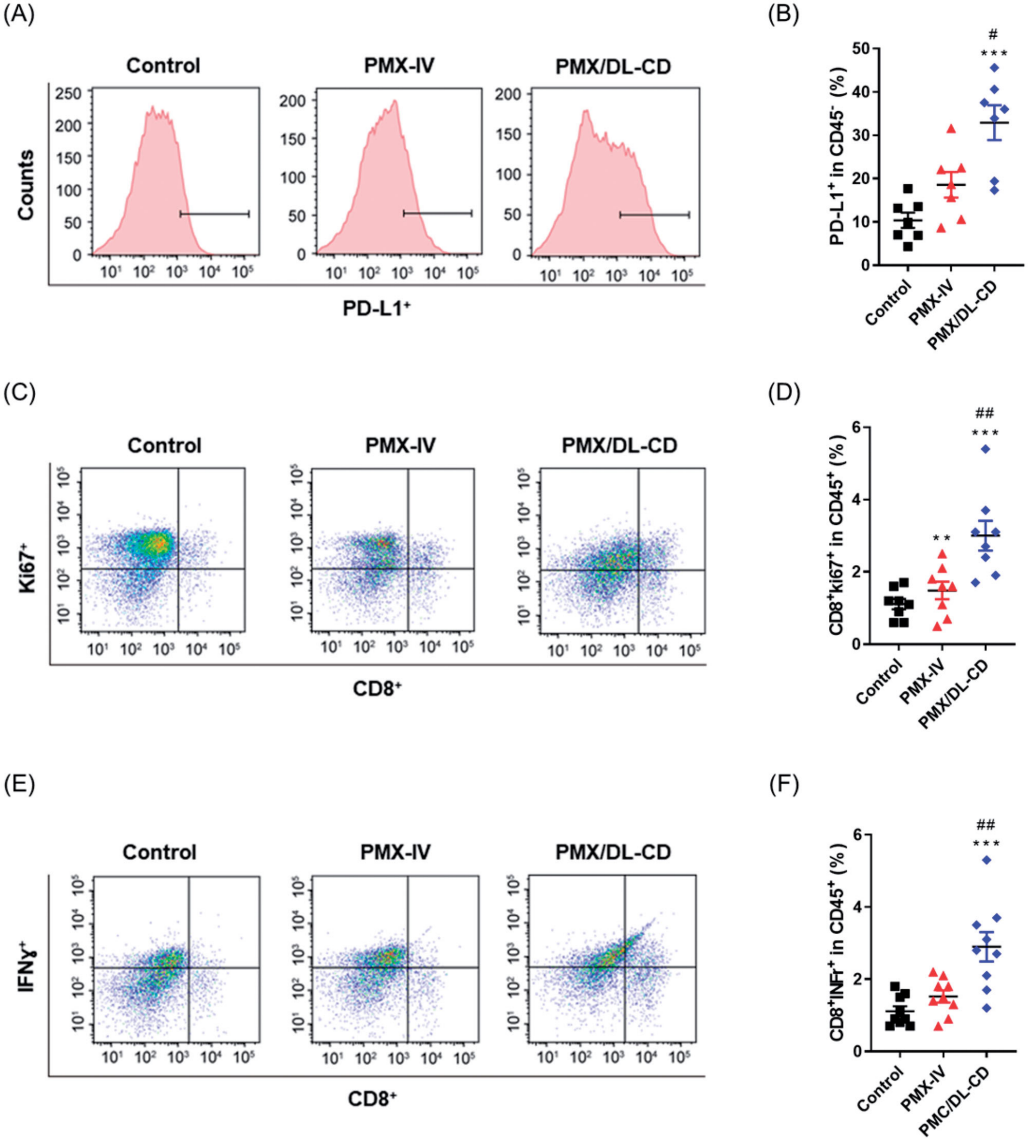
(A) PD-L1 expression in tumor cells measured using flow cytometry and (B) quantification thereof in the control, MTD, and MCT groups. (C) Gating of CD45^+^CD8^+^Ki67^+^ T cells using flow cytometry and (D) quantification thereof. (E) Characterization of CD45^+^CD8^+^IFN-γ^+^ T cells using flow cytometry and (F) quantification thereof. All values are means ± SEMs (*n* = 5). **p < .01, and ***p < .001 compared to untreated controls; ^#^*p* < .05, and ^##^*p* < .01 compared to the PMX-IV group.

We also evaluated whether PMX treatment-induced PD-L1 expression in tumor cells modulates tumor immunity. We found that PD-L1 expression in PMX-IV and PMX/DL-CD groups was two and three times greater compared to the control group, respectively (Figure 5(A,B)). This suggests that PMX treatment directly or indirectly increases PD-L1 expression. We further investigated the suppression of T-cell functions, such as proliferative capability and cytokine release, by PMX treatment. Oral PMX MCT significantly enhanced CD8^+^ T-cell proliferation compared to the PMX MTD and control groups (Figure 5(C,D)). Similarly, PMX MCT treatment substantially increased the IFN-γ-releasing CD8^+^ T-cell population compared to the control group (Figure 5(E,F)). These data demonstrated that MCT treatment using PMX not only enhanced lymphocyte infiltration but also suppressed T-cell function.

**Figure 6.**
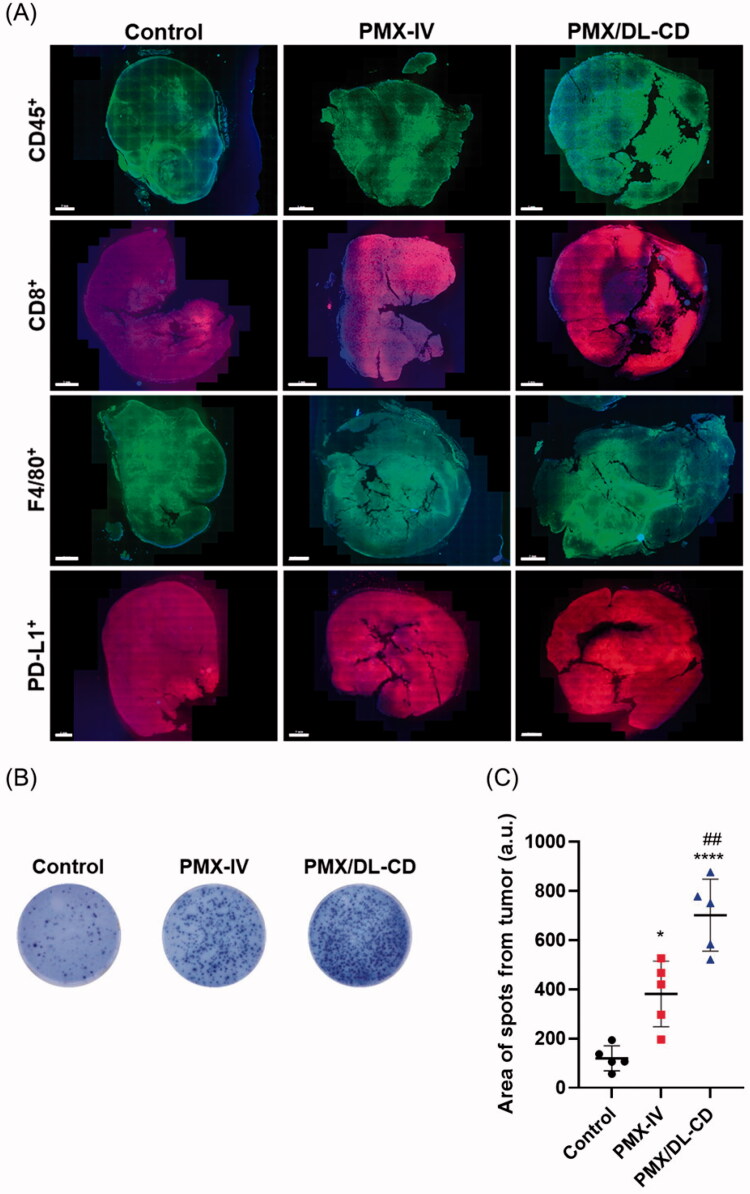
(A) Staining of tumor-infiltrating lymphocytes, including CD45^+^, CD8^+^, and F4/80^+^ cells, and PD-L1, in tumor tissues. Scale bar: 2 mm. (B) ELISPOT of IFN-γ after stimulation of tumor lymphocytes with tumor-specific antigens in each group. (C) Quantification results. All values are means ± SEMs (*n* = 5). *p < .05, ****p < .0001 compared to the untreated control; ^##^*p* < .01 compared to the PMX-IV group.

These results were also demonstrated by slide staining and ELISPOT of tumor-infiltrating lymphocytes. Whole slide images of CD45^+^, CD8^+^, and PD-L1^+^ cells were consistent with the flow cytometry findings (Figure 6(A)). We also found that the populations of F4/80^+^ cells, the tumor-infiltrating macrophages, were substantially elevated in the PMX/DL-CD group compared to the control and PMX-IV groups (Figure 6(A)). IFN-γ-specific ELISPOT also showed that PMX/DL-CD was most effective in eliciting tumor-specific immunity among the three groups. These data demonstrated that PMX/DL-CD effectively boosts the tumor-specific immunity of tumor-infiltrating lymphocytes (Figure 6(B,C)).

Collectively, these data suggest that metronomic oral PMX treatment is more effective for tumor-specific immunity enhancement because of increased expression of lymphocytes in tumor tissue and suppression of tumor-specific immunity. These data also indicate that metronomic oral administration of PMX has a synergistic effect with ICIs.

### 3.8. Synergistic antitumor effects of combined treatment with metronomic oral PMX/DL-CD and aPD-1 in CT26.CL25 cell-bearing mice

**Figure 7.**
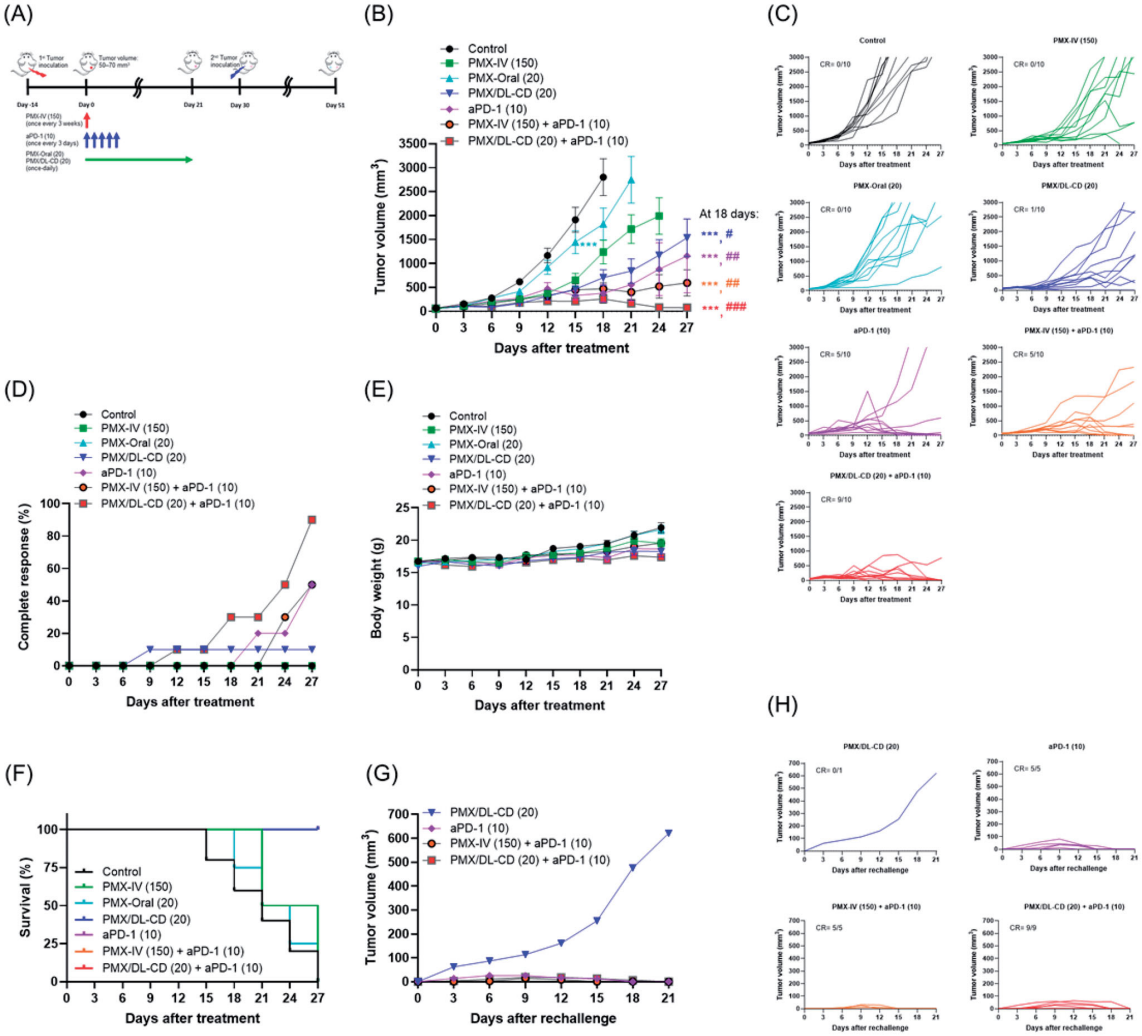
Tumor regression and memory responses elicited by once every three weeks, intravenous (IV) injections of 150 mg/kg PMX [PMX-IV (150)] for 21 days; once-a-day oral administration of aqueous solution of 20 mg/kg PMX [PMX-Oral (20)] or PMX/DL(1:1)-CD as 20 mg/kg PMX [PMX/DL-CD (20)] for 21 days; once every three days, intraperitoneal injection of 10 mg/kg aPD-1 [aPD-1 (10)] for 14 days; co-administration of PMX-IV (150) for 21 days and aPD-1 (10) for 14 days [PMX-IV (150) + aPD-1 (10)]; and co-administration of oral PMX/DL-CD (20) for 21 days and aPD-1 (10) for 14 days [PMX/DL-CD (20) + aPD-1 (10)], respectively, in the CT26.CL25 tumor-bearing mice. (A) Schematic illustration of schedules for drug administration and rechallenge; (B) tumor growth; (C) individual tumor volume; (D) complete response; (E) changes in body weight; and (F) survival rates for each group; (G) tumor volume in the PMX/DL-CD (20), aPD-1 (10), PMX-IV (150) + aPD-1 (10), and PMX/DL-CD (20) + aPD-1 (10) groups after rechallenging with CT26.CL25 cells to the mice having completely regressed tumors; (H) individual tumor volume in each group. All values represent means ± SEMs (*n* = 10 per group). ***p < .001 compared to the control group; ^#^*p* < .05, ^##^*p* < .01, and ^###^*p* < .001 compared to the PMX-Oral (20) group.

To investigate a synergistic antitumor efficacy of oral PMX/DL-CD in combination with aPD-1 using immunomodulatory effects of metronomic scheduling of PMX, we performed an *in vivo* combination study with aPD-1 in the CT26.CL25 tumor model (Figure 7(A)). Tumors in mice treated with PMX-IV (150) started to grow significantly 15 days after the injections (Figure 7(B)). Tumor growth was effectively suppressed with oral PMX/DL-CD (20) for 21 days, resulting in 74.8 and 61.4% maximal tumor growth inhibitions up to day 18, compared to the control and PMX-Oral (20) groups, respectively (Figure 7(B,C)). However, the remaining tumor tissues grew again quickly, soon after PMX/DL-CD (20) was stopped on day 21. Tumor growth in mice treated with aPD-1 alone was also significantly delayed up to day 18; however, the remaining tumor volume increased continuously, and only 50% of mice showed a CR (Figure 7(C,D)). On the other hand, a combination of PMX-IV (150) and aPD-1 (10) exhibited substantial anticancer effects, leading to 73.9 and 48.7% greater tumor-growth delays, respectively, compared to the mice treated with PMX-IV (150) or aPD-1 (10) alone (Figure 7(B,C)). However, continuous tumor growth was observed in 50% of mice, with no significant improvement of incomplete rejection (Figure 7(D)). Co-administration of oral PMX/DL-CD (20) and aPD-1 (10) generated drastically improved anticancer effects without significant toxicity and resulted in 90% of mice being tumor-free (Figure 7(B–E)). Furthermore, 100% of mice were alive in the oral PMX/DL-CD (20) and aPD-1 (10) group at 27 days after drug treatment, which was longer than the control and PMX-IV (150) groups (10 and 50% for each group, respectively) (Figure 7(F)).

To investigate further the effect of oral PMX/DL-CD (20) and aPD-1 (10) combination on memory T-cell response *in vivo*, the mice that showed a CR were rechallenged with CT26.CL25 cells at 30 days after initial treatment. The rechallenged tumor in one mouse treated with PMX/DL-CD (20) developed and showed continuous tumor growth without drug administration, indicating no memory immune response (Figure 7(G,H)). Meanwhile, all rechallenged tumors in the mice treated with aPD-1 (10) alone, or in combination with PMX-IV (150) or PMX/DL-CD (20), were completely rejected up to day 21 (Figure 7(G,H)). These results suggest that the combined treatment with PMX/DL-CD (20) and aPD-1 (10) can provide durable antitumor effects by generating immunological memory.

## 4. Conclusion

Metronomic PMX treatment is an effective alternative for achieving the synergistic effects of combination treatment, compared to MTD treatment. However, there has been controversy regarding oral delivery of PMX for frequent metronomic dosing. In this study, we developed colloidal formulations of oral PMX (PMX/DL-CD) and demonstrated adequate absorption. We found that treatment with PMX/DL-CD successfully induced ICD, which resulted in the release of danger signals, such as HMGB-1 and CRT. We also demonstrated that low-dose metronomic oral PMX treatment was associated with increased numbers of tumor-infiltrating lymphocytes and enhanced T cell function, compared to control and PMX-IV groups. Furthermore, oral PMX/DL-CD elicited stronger antitumor effects in combination with aPD-1 antibodies compared to the PMX-IV group, indicating that metronomic oral PMX has stronger immune-supportive effects than IV MTD treatment. Our findings suggest that metronomic oral PMX has the potential for use in combination with immunotherapy to elicit synergistic antitumor effects with less toxicity.

## Supplementary Material

Supplemental Material
