# Supplementary material for: Metronomic delivery of orally available pemetrexed-incorporated colloidal dispersions for boosting tumor-specific immunity
Source: Drug Deliv. 2021 Nov 3;28(1):2313–28. doi: 10.1080/10717544.2021.1995077 (PMC8567874; doi:10.1080/10717544.2021.1995077)
Supplement: Supplemental Material [file IDRD_A_1995077_SM4663.docx]

**Supporting Information**

**Metronomic delivery of orally available pemetrexed-incorporated colloidal dispersions for boosting tumor-specific immunity**

Ruby Maharjan^a^*, Laxman Subedi^b^*, Rudra Pangeni^c^, Saurav Kumar Jha^b^, Seo Hee Kang^d^, Kwan-Young Chang^d^, Youngro Byun^a^, Jeong Uk Choi^e^, Jin Woo Park^b,f^

^a^Research Institute of Pharmaceutical Sciences, College of Pharmacy, Seoul National University, Seoul 08826, Republic of Korea

^b^Department of Biomedicine, Health & Life Convergence Sciences, BK21 Four, Biomedical and Healthcare Research Institute, Mokpo National University, Jeonnam 58554 Republic of Korea

^c^Department of Pharmaceutics, School of Pharmacy, Virginia Commonwealth University, VA 23298, USA

^d^Global R&D center, IcureBNP, Seoul 08511, Republic of Korea

^e^College of Pharmacy, Chonnam National University, Gwangju 61186, Republic of Korea

^f^College of Pharmacy and Natural Medicine Research Institute, Mokpo National University, Jeonnam 58554, Republic of Korea

*Ruby Maharjan and Laxman Subedi contributed equally to this work as first authors.

**Addresses for correspondences:** Jeong Uk Choi, Email: cju0667@jnu.ac.kr, Department of Pharmacy, College of Pharmacy, Chonnam National University, 77 Yongbong-ro, Buk-gu, Gwangju, 61186, Republic of Korea; Jin Woo Park, Email: [jwpark@mokpo.ac.kr](mailto:jwpark@mokpo.ac.kr), Department of Pharmacy, College of Pharmacy, Mokpo National University, 1666 Youngsan-ro, Muan-gun, Jeonnam 58554, Republic of Korea

**Running head:** Oral metronomic delivery of pemetrexed to enhance cancer immunotherapy

**Supplemental data**

**Table S1.** Apparent partition coefficients of different PMX forms.

| Test material | Log P^a^ |
| --- | --- |
| PMX | −1.14 ± 0.01 |
| PMX/DL(1:1) | 0.04 ± 0.01 |
| PMX/DL(1:2) | 1.24 ± 0.02 |
| PMX/DL(1:1)-CD | 0.08 ± 0.03 |
| PMX/DL(1:2)-CD | 0.58 ± 0.03 |

^a^Logarithm of the octanol/water partition coefficient. Each value represents the mean ± standard deviation (*n* = 4).

**Table S2.** Outlet concentration of PMX (C_out_), perfusate exit flow (Q_out_), and corrected outlet PMX concentration (C_out,corr_) in the *in situ* rat intestinal perfusion study.

| Test material | Time (min) | 15 | 30 | 45 | 60 | 75 | 90 | 105 | 120 |
| --- | --- | --- | --- | --- | --- | --- | --- | --- | --- |
| PMX | C_out_ (μg/mL) | 93.1 ± 1.73 | 93.2 ± 0.04 | 92.5 ± 0.51 | 94.9 ± 0.19 | 94.7 ± 1.19 | 95.3 ± 0.87 | 94.7 ± 1.92 | 94.7 ± 1.82 |
|  | Q_out_ (mL/min) | 0.202 ± 0.002 | 0.206 ± 0.003 | 0.204 ± 0.002 | 0.201 ± 0.000 | 0.203 ± 0.001 | 0.203 ± 0.001 | 0.203 ± 0.004 | 0.204 ± 0.001 |
|  | C_out, corr_ (μg/mL) | 93.8 ± 2.73 | 96.0 ± 1.36 | 94.1 ± 0.46 | 95.4 ± 0.19 | 95.8 ± 0.87 | 96.5 ± 0.54 | 95.8 ± 0.27 | 96.4 ± 1.52 |
| PMX/DL(1:1) | C_out_ (μg/mL) | 71.9 ± 10.1 | 76.6 ± 9.08 | 78.1 ± 7.43 | 79.3 ± 5.30 | 81.2 ± 6.03 | 81.9 ± 2.57 | 82.7 ± 2.66 | 83.8 ± 0.54 |
|  | Q_out_ (mL/min) | 0.230 ± 0.014 | 0.222 ± 0.013 | 0.215 ± 0.001 | 0.215 ± 0.003 | 0.217 ± 0.005 | 0.215 ± 0.000 | 0.214 ± 0.000 | 0.218 ± 0.004 |
|  | C_out, corr_ (μg/mL) | 82.3 ± 6.52 | 84.5 ± 4.92 | 83.8 ± 7.70 | 85.2 ± 4.57 | 87.8 ± 4.52 | 88.0 ± 2.77 | 88.5 ± 2.85 | 91.3 ± 2.37 |
| PMX/DL(1:2) | C_out_ (μg/mL) | 74.5 ± 8.07 | 76.6 ± 8.81 | 77.9 ± 7.88 | 79.5 ± 8.57 | 79.5 ± 10.8 | 82.9 ± 6.83 | 83.3 ± 7.04 | 84.5 ± 7.38 |
|  | Q_out_ (mL/min) | 0.221 ± 0.017 | 0.217 ± 0.017 | 0.217 ± 0.018 | 0.212 ± 0.010 | 0.214 ± 0.018 | 0.206 ± 0.003 | 0.205 ± 0.006 | 0.206 ± 0.004 |
|  | C_out, corr_ (μg/mL) | 81.9 ± 3.06 | 82.7 ± 3.91 | 83.9 ± 1.89 | 84.0 ± 5.21 | 84.6 ± 4.90 | 85.2 ± 5.80 | 85.4 ± 4.74 | 87.1 ± 6.12 |
| PMX/DL(1:1)-CD | C_out_ (μg/mL) | 75.7 ± 15.0 | 72.8 ± 5.62 | 73.9 ± 7.22 | 76.8 ± 12.3 | 69.5 ± 3.42 | 70.9 ± 6.44 | 67.6 ± 2.04 | 70.1 ± 5.40 |
|  | Q_out_ (mL/min) | 0.208 ± 0.010 | 0.208 ± 0.008 | 0.207 ± 0.010 | 0.207 ± 0.006 | 0.207 ± 0.005 | 0.208 ± 0.008 | 0.207 ± 0.005 | 0.206 ± 0.006 |
|  | C_out, corr_ (μg/mL) | 78.3 ± 11.9 | 75.4 ± 3.00 | 76.3 ± 3.82 | 79.2 ± 10.2 | 71.7 ± 1.82 | 73.4 ± 3.92 | 69.8 ± 0.43 | 71.9 ± 3.32 |
| PMX/DL(1:2)-CD | C_out_ (μg/mL) | 64.7 ± 15.8 | 65.7 ± 15.5 | 66.2 ± 15.9 | 64.6 ± 17.4 | 67.7 ± 17.3 | 67.3 ± 15.6 | 68.1 ± 14.9 | 69.4 ± 13.3 |
|  | Q_out_ (mL/min) | 0.218 ± 0.020 | 0.209 ± 0.008 | 0.211 ± 0.011 | 0.216 ± 0.021 | 0.211 ± 0.014 | 0.209 ± 0.012 | 0.208 ± 0.011 | 0.204 ± 0.006 |
|  | C_out, corr_ (μg/mL) | 69.9 ± 13.1 | 68.2 ± 13.9 | 69.3 ± 13.4 | 68.9 ± 13.9 | 70.7 ± 14.5 | 70.0 ± 13.6 | 70.3 ± 13.0 | 70.5 ± 12.3 |

PMX, pemetrexed; DL, deoxycholic acid-lysine conjugate; PMX/DL(1:1) and PMX/DL(1:2), ion-pairing complex of PMX with DL in 1:1 or 1:2 molar ratios, respectively; CD, colloidal dispersion; PMX/DL(1:1)-CD and PMX/DL(1:2)-CD, CD of PMX/DL(1:1) or PMX/DL(1:2) formed by Kolliphor P188 and Labrasol, respectively; C_out_, outlet concentration of PMX; Q_in_, flow rate entering the intestine; Q_out_, perfusate exit flow (net weight/15 min, assumed density of 1.0 g/mL) for the specified time interval. C_out,corr_, corrected outlet PMX concentration; C_out,corr_ = C_out_ × (Q_out_/Q_in_).


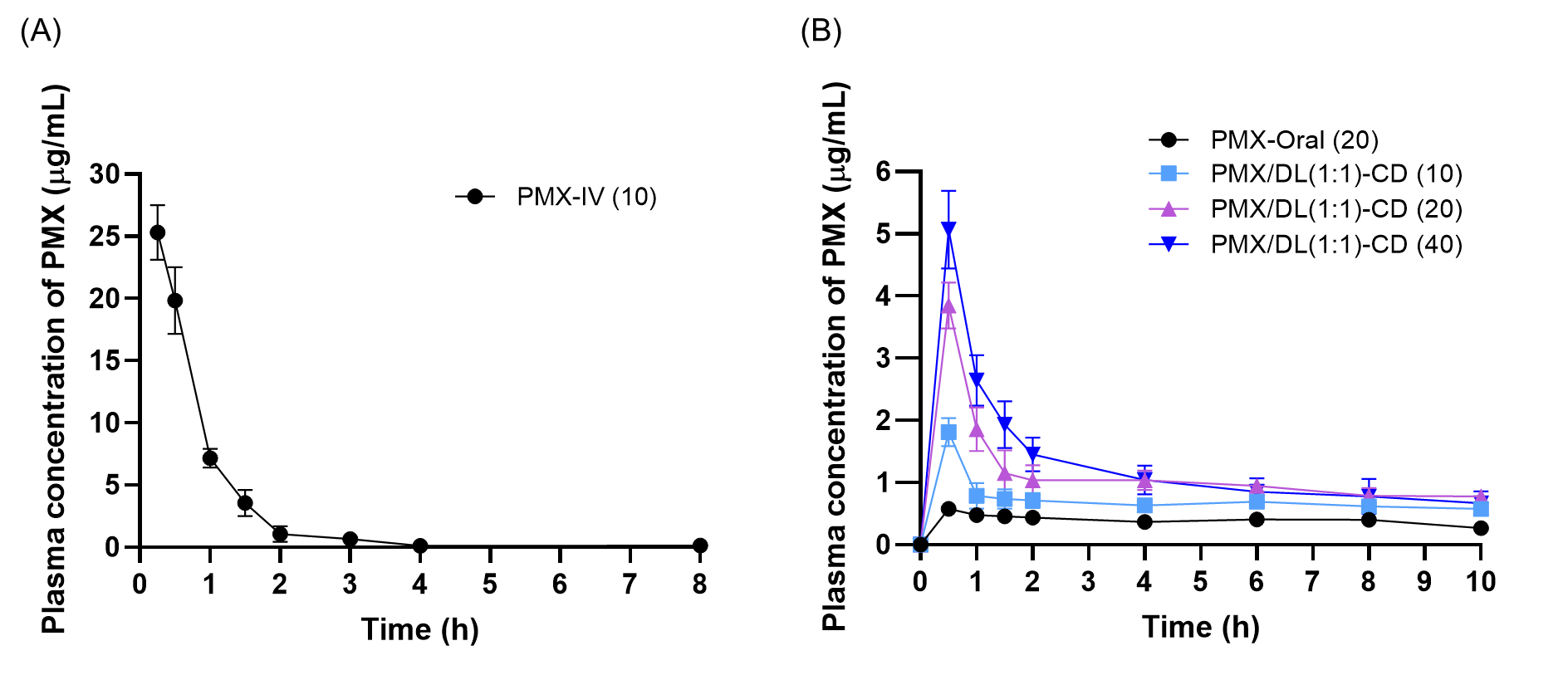


**Figure S1.** Mean plasma concentration-time profiles of pemetrexed (PMX) in rats. (A) Mean plasma concentration-time profile of PMX after a single intravenous injection of 10 mg/kg PMX [PMX-IV (10)] (B) Mean plasma concentration-time profiles of PMX after a single oral administration of 20 mg/kg PMX [PMX-Oral (20)], PMX/DL(1:1)-CD equivalent to 10 mg/kg PMX [PMX/DL(1:1)-CD (10)], PMX/DL(1:1)-CD equivalent to 20 mg/kg PMX [PMX/DL(1:1)-CD (20)], and PMX/DL(1:1)-CD equivalent to 40 mg/kg PMX [PMX/DL(1:1)-CD (40)]. All values are means ± SDs (*n* = 4).
